# Effect of scaling and root planing on root canal-treated teeth with periodontal pockets

**DOI:** 10.6026/973206300211952

**Published:** 2025-07-31

**Authors:** Ankur Singh Rajpoot, Deepika Baghel, Varsha Choubey, Jasleen Kaur, Disha Gupta, Rohit Bansal, Anukriti Kumari

**Affiliations:** 1 Department of Periodontology, RKDF Dental College and Research Centre, Bhopal, Madhya Pradesh, India; 2 Department of Dentistry, Institute of Dental Education and Advance Studies, Gwalior, Madhya Pradesh, India; 3 Department of Periodontology, Hitkarini Dental College and Hospital, Jabalpur, Madhya Pradesh, India; 4 Department of Dentistry, Pt. Bhagwat Dayal Sharma Post Graduate Institute of Dental Science, Rohtak, Haryana, India; 5 Department of Conservative Dentistry and Endodontics, SGT Dental College, Hospital & Research Institute, SGT University, Gurugram, Haryana, India; 6 Department of Conservative Dentistry and Endodontics, PDM University, Bahadurgarh, Haryana, India; 7 Department of Oral Medicine and Radiology, School of Dental Sciences, Sharda University, Greater Noida, India

**Keywords:** Clinical attachment level (CAL), probing pocket depth (PPD), root canal treatment (RCT), scaling and root planing (SRP), tooth mobility

## Abstract

The effect of scaling and root planing (SRP) on the success of root canal-treated teeth with periodontal pockets is of interest. A randomized controlled trial with 100 adult patients was conducted, with one group receiving SRP and the other receiving traditional periodontal treatment. Clinical outcomes, including probing pocket depth (PPD), clinical attachment level (CAL), bleeding on probing (BOP) and tooth mobility, were assessed over six months. The SRP group showed significant improvement in all clinical parameters compared to the control group. Thus, SRP improves the long-term success of root canal-treated teeth, particularly in patients with periodontal disease.

## Background:

Scaling and root planing (SRP) is a basic non-surgical periodontal treatment applied to treat periodontal disease [1]. When periodontal disease affects teeth that have undergone root canal treatment, the dynamics of therapy get more complex since the result of the root canal treatment could rely on the periodontal state of the tooth [2]. Root canal therapy (RCT) is a well-established technique used to treat infections or inflammation in the pulp of a tooth by eliminating the disease and sealing the root canals [3]. Still, teeth undergoing root canals could be sensitive to periodontal issues, including the development of periodontal pockets [4]. Periodontal pockets, which develop between the gum and the tooth from the breakdown of supporting tissues, can create extra difficulties if not properly maintained. Such pits in teeth undergoing root canals raise issues about the long-term survival and prognosis of these teeth [5]. RCT aims to clear the infection within the tooth; therefore, incorrect treatment of periodontal disease can affect the outcome of the surgery [6]. The periodontal tissue surrounding a tooth that has had root canal therapy determines mostly whether a tooth maintains its general health and stability [7]. Periodontal pockets create an environment that promotes bacterial invasion, which can compromise the tooth that has been root canal-treated and lead to reinfection, more bone loss, or perhaps tooth movement. This two-fold focus on endodontic and periodontal health requires a comprehensive therapy plan that simultaneously treats both conditions [8]. Thus, the general state of root canal-treated teeth, including periodontal pockets, can be much influenced by scaling and root planing. By reducing the bacterial burden and supporting periodontal tissues to heal, SRP is most likely to increase the success rate of root canal treatments [9]. Still under significant debate in the dental field is the connection between SRP and the success of root canal-treated teeth with periodontal pockets. Although some studies indicate the problems resulting from significant periodontal pockets around the root canal-treated area, others hypothesise that SRP may enhance RCT results by reducing the likelihood of reinfection and aiding periodontal healing [10]. Understanding the interplay between endodontic and periodontal tissues will help one predict the success of teeth treated with a root canal that contain periodontal pockets. SRP is hence really important [11]. Clinically, SRP has important effects on either preserving or improving the state of these teeth. Through the prediction of periodontal disease, one can accurately assess the success of root canal procedures, thereby guiding treatment preparation and enhancing patient outcomes [12]. Therefore, it is of interest to describe how SRP influences the long-term success of root canal-treated teeth with periodontal pockets and its potential incorporation into treatment protocols for better clinical outcomes.

## Methodology:

The effects of SRP included in periodontal disease treatment in teeth treated in a root canal were evaluated using a prospective, randomised controlled clinical trial design. One hundred adult patients, ranging in age from 18 to 70, who had received root canal therapy, had at least one tooth with periodontal pockets of 4mm or more. Every participant was randomly assigned to either Group A (SRP group) or Group B (control group). Group A comprised 50 patients undergoing SRP treatment for the periodontal pockets in teeth repaired by root canal; Group B consisted of 50 patients receiving traditional periodontal treatment free of SRP. Following up on appointments set for one month, three months and six months after treatment, both groups were advised on maintaining good dental hygiene. Group A had local anaesthetic SRP from the periodontal pockets and root surfaces, therefore removing calculus, plaque and biofilm. Careful design of the root surfaces helped to lower bacterial burden and stimulate surrounding periodontal repair. Group B received no root planning but underwent basic periodontal treatment, which included professional cleaning and dental hygiene advice. Clinically evaluated at baseline and at follow-up visits was Probing Pocket Depth (PPD), Clinical Attachment Level (CAL), bleeding on Probing (BOP), tooth movement and radiography review. Additionally, a self-reported questionnaire was used to evaluate patient satisfaction with the therapeutic results. Data were analysed using statistical instruments; paired t-tests were used to examine variations across each group over time. Independent t-tests were used for continuous data and chi-square tests were used for categorical variables to facilitate between-group comparisons. The institutional review board approved the research plan and ethical issues were resolved, as each person signed an informed consent form. Notwithstanding the obvious limitations-variability in patient reactions, non-compliance with follow-up visits and a small sample size-the method aimed to provide insight into how SRP affected root canal-treated teeth with periodontal pockets. This study is therefore significant in clarifying how SRP might influence the long-term efficacy of root canal-treated teeth in patients with periodontal disease, thereby providing a more comprehensive approach to dental treatment.

## Results:

The results of this study were evaluated based on the clinical parameters measured at baseline and follow-up visits at 1, 3 and 6 months. The main clinical parameters evaluated were Probing Pocket Depth (PPD), Clinical Attachment Level (CAL), bleeding on Probing (BOP) and tooth mobility. Additionally, radiographic evaluation was used to assess changes in bone levels around root canal-treated teeth. Data analysis showed significant improvements in periodontal health for the SRP group (Group A) compared to the control group (Group B). A reduction in Probing Pocket Depth (PPD) was observed in both groups over time. However, the SRP group demonstrated a more significant reduction compared to the control group. At baseline, the mean PPD for Group A was 6.2mm and for Group B, it was 6.1mm. At the 6-month follow-up, Group A showed a significant reduction to 3.5mm, while Group B only reduced to 5.0mm (Table 1 - see PDF). The Clinical Attachment Level (CAL) showed improvement in both groups; however, the SRP group demonstrated greater improvement. At baseline, the mean CAL for Group A was 5.0mm and for Group B, it was 5.2mm. At the 6-month follow-up, Group A improved to 2.6mm, while Group B only improved to 4.3mm (Table 2 - see PDF). Bleeding on Probing (BOP) was significantly reduced in the SRP group compared to the control group. At baseline, 70% of sites in Group A and 72% of sites in Group B showed BOP. By the 6-month follow-up, the BOP was reduced to 28% in Group A, while in Group B, it decreased to 48% (Table 3 - see PDF). Tooth mobility was assessed using a periodontal probe. In both groups, there was minimal change in tooth mobility, but the SRP group showed a slight improvement. At baseline, Group A had a mobility score of 1.2 (on a scale of 0 to 3) and Group B had a mobility score of 1.1. At the 6-month follow-up, Group A's mobility score reduce to 0.8, while Group B's mobility score remained at 1.0 (Table 4 - see PDF). Radiographic evaluation showed a significant improvement in bone levels around the root canal-treated teeth in Group A compared to Group B. At baseline, there was no significant difference in bone levels between the groups. However, after 6 months, Group A showed a noticeable increase in bone density, while Group B showed minimal change. Patient satisfaction was assessed through a self-reported questionnaire, where Group A reported higher levels of satisfaction compared to Group B. 85% of patients in Group A were satisfied with the treatment outcomes, compared to 65% in Group B. Paired t-tests showed statistically significant reductions in PPD, CAL and BOP in Group A compared to Group B (p < 0.05). The independent t-tests for between-group comparisons further confirmed that Group A had better clinical improvements than Group B across all measured parameters (Table 5 - see PDF).

## Discussion:

This paper demonstrates how much the periodontal health of teeth rebuilt following a root canal with periodontal pockets improves by scaling and root planing (SRP). Reductions in Probing Pocket Depth (PPD), increases in Clinical Attachment Level (CAL) and a decline in Bleeding on Probing (BOP) all point to SRP as a really useful additional treatment. These findings align with the growing body of evidence, suggesting that, most critically, the long-term efficacy of teeth treated with root canals is influenced by the state of the periodontium. Better patient satisfaction and radiographic changes, which replace conventional periodontal treatments, demonstrate even more SRP's therapeutic effectiveness. These findings align with a study by Cugini *et al.* (2000) [13], which agree with earlier studies on the effect of SRP on root canal-treated teeth with periodontal disease. Reflecting the results of the current research, SRP drastically reduced PPD and raised CAL. Cugini's research primarily focused on periodontal measurements, which showed ongoing improvements, suggesting that SRP has long-lasting effects on the long-term benefits. Apart from reducing periodontal pockets, a 2024 study by Duraisamy *et al.* [14] also showed that SRP enhanced the clinical results of teeth restored following root canal treatment. In addressing teeth with both endodontic and periodontal issues, Duraisamy study concentrated on the combined benefits of SRP and root canal treatment. The changes in BOP seen in their research aligned with our findings in that SRP reduced periodontal tissue inflammation and bleeding. In 2021, Fang *et al.* [15] further supported the benefits of SRP in root canal-treated teeth with periodontal disease, as SRP enhanced both clinical and radiographic results. Lee *et al.* underscored the positive link between better results for teeth treated with root canal therapy and enhanced periodontal condition in keeping with the conclusions of this study. The radiographic changes observed in both studies naturally highlight the need to treat endodontic and periodontal problems in line with the course.

## Conclusion:

This study showed that SRP significantly improved periodontal health in root canal-treated teeth, with reductions in Probing Pocket Depth, better Clinical Attachment Levels and decreased Bleeding on Probing. SRP also resulted in higher patient satisfaction and radiographic improvements compared to conventional treatment. Therefore, SRP is an effective adjunctive therapy for enhancing the long-term success of root canal-treated teeth with periodontal disease.
